# Video Game Playing Is Independently Associated with Blood Pressure and Lipids in Overweight and Obese Adolescents

**DOI:** 10.1371/journal.pone.0026643

**Published:** 2011-11-01

**Authors:** Gary S. Goldfield, Glen P. Kenny, Stasia Hadjiyannakis, Penny Phillips, Angela S. Alberga, Travis J. Saunders, Mark S. Tremblay, Janine Malcolm, Denis Prud'homme, Rejeanne Gougeon, Ronald J. Sigal

**Affiliations:** 1 Healthy Active Living and Obesity Research Group, Children's Hospital of Eastern Ontario Research Institute, Ottawa, Ontario, Canada; 2 School of Human Kinetics, Faculty of Health Sciences, University of Ottawa, Ottawa, Ontario, Canada; 3 Department of Pediatrics, University of Ottawa, Ottawa, Ontario, Canada; 4 School of Psychology, University of Ottawa, Ottawa, Ontario, Canada; 5 Department of Psychology, Carleton University, Ottawa, Ontario, Canada; 6 Ottawa Hospital Research Institute, Ottawa, Ontario, Canada; 7 Department of Nutrition, McGill University, Montreal, Québec, Canada; 8 Department of Medicine, Cardiac Sciences and Community Health Sciences, Faculties of Medicine and Kinesiology, University of Calgary, Calgary, Alberta, Canada; I2MC INSERM UMR U1048, France

## Abstract

**Objective:**

To examine the association between duration and type of screen time (TV, video games, computer time) and blood pressure (BP) and lipids in overweight and obese adolescents.

**Design:**

This is a cross-sectional study of 282 overweight or obese adolescents aged 14–18 years (86 males, 196 females) assessed at baseline prior to beginning a lifestyle intervention study for weight control. Sedentary behaviours, defined as hours per day spent watching TV, playing video games, recreational computer use and total screen time were measured by self-report. We examined the associations between sedentary behaviours and BP and lipids using multiple linear regression.

**Results:**

Seated **v**ideo gaming was the only sedentary behaviour associated with elevated BP and lipids before and after adjustment for age, sex, pubertal stage, parental education, body mass index (BMI), caloric intake, percent intake in dietary fat, physical activity (PA) duration, and PA intensity. Specifically, video gaming remained positively associated with systolic BP (adjusted r = 0.13, β = 1.1, p<0.05) and total cholesterol/HDL ratio (adjusted r = 0.12, β = 0.14, p<0.05).

**Conclusions:**

Playing video games was the only form of sedentary behaviour that was independently associated with increased BP and lipids. Our findings provide support for reducing time spent playing seated video games as a possible means to promote health and prevent the incidence of cardiovascular disease (CVD) risk factors in this high risk group of overweight and obese adolescents. Future research is needed to first replicate these findings and subsequently aim to elucidate the mechanisms linking seated video gaming and elevated BP and lipids in this high risk population.

**Trial Registration:**

Clinicaltrials.gov NCT00195858

## Introduction

Most adolescents living in Western countries spend excessive amounts of time being sedentary, mainly in the form of screen time behaviours such as TV viewing, seated video gaming, and recreational computer use [1]–[3]. This is concerning because sedentary behaviours track throughout adolescence and into adulthood [4], and sedentariness via screen time in adulthood is associated with increased morbidity and mortality, making the relationship between sedentary behaviour and health indicators an important area of study [5]. Many of the studies conducted in the pediatric population have focused primarily on glucose-related measures with relatively little attention to traditional cardiovascular disease (CVD) risk factors such as high blood pressure (BP) and dyslipidemia. However, there is evidence that dyslipidemia and elevated BP are being increasingly observed in youth, especially overweight and obese youth [6], [7], and that these CVD risk factors in adolescence predict the development of CVD and mortality in adulthood [8], [9]. Moreover, animal research has shown that extended bouts of sedentary behaviour result in dramatic reductions in lipoprotein lipase activity leading to reductions in high-density lipoprotein cholesterol (HDL-C) in rat skeletal muscle [10]. Similarly, excessive sedentary behaviour is associated with increases in low density lipoprotein cholesterol (LDL-C) and triglycerides in adult humans [11].

In the studies focusing on BP and lipids as an indicator of CVD risk in youth, screen time was independently associated with increased CVD risk after adjusting for moderate-to-vigorous intensity physical activity (PA) and other confounders in some studies [12]–[14] but not others [15]–[17]. However, the studies demonstrating null findings have design limitations including limited statistical power [16], TV viewing as the only index of sedentary behaviour [15], a limited number of measured CVD risk factors [15], and the assessment of screen time not including video gaming [17]. Including seated video gaming as part of screen time measurement is important because it is appealing to adolescents [18] and is associated with obesity in youth [19]–[21], possibly due to increased food intake [22], [23]. Moreover, recent research found that different types of screen behaviour may have different and independent effects on chronic disease risk in youth [24], [25] but again these studies failed to include video gaming as part of screen time. Thus, measurement of time spent playing video games in association with the full spectrum of BP and lipid measurements as indicators of CVD risk factors remains unclear.

The purpose of this study was to examine the independent relationships between the volume and types of sedentary screen time behaviours (i.e., TV viewing, seated video games, computer time) with systolic and diastolic BP and lipids in overweight and obese adolescents, controlling for a wide range of confounders.

## Materials and Methods

### Participants

The sample consisted of 282 adolescents who were either obese (≥95 body mass index (BMI) percentile for age and sex) or overweight (85^th^ to 94^th^ BMI percentile for age and sex) based on cut-off values from the Centers for Disease Control (CDC) growth charts [26], recruited as potential participants in a diet and exercise trial. Those participants who were overweight had to have at least one comorbid CVD or diabetes risk factor, including family history, to be included in the study. The sample was comprised of 86 males and 196 female aged 14 to 18 years, with a mean age of 15.5±1.4 years. All participants were Tanner stage IV or V with respect to pubertal development, and the mean BMI was 34.4±4.3 kg/m^2^. The majority (70%) of the sample was Caucasian, 12% were African Canadian, 2% Asian, 3% Hispanic, 2% First Nations, 4% Arabic, 4% mixed race, and 3% categorized as other. Parents of participants were well educated, with 75% of mothers and 68% of fathers having completed some university or community college program. Participants were recruited by posters and advertisements in the Children's Hospital of Eastern Ontario's endocrine/obesity clinic, by radio and bus advertisements, and flyers in community physicians' offices. Bus advertisements were the most effective recruitment strategy, accounting for 48% of recruitment. Data for this analysis come from the Healthy Eating and Aerobic and Resistance Training in Youth (HEARTY) trial, a randomized controlled exercise intervention aimed at reducing adiposity in obese adolescents. The data reported herein represent baseline data collected before the intervention from 2005 to 2010. Of the 358 adolescents, 282 (79%) had complete data for analysis from the baseline data collection of the HEARTY trial. All participants completed the testing individually (i.e. one on one) with the research coordinator.

### Ethics Statement

Informed written consent from each participant was obtained in accordance with the principles expressed in the Declaration of Helsinki guidelines for human subjects. In the case of minors, written informed consent was also obtained by parents or legal guardians. This study was reviewed and approved by the Research Ethics Boards of the Children's Hospital of Eastern Ontario (CHEO) and The Ottawa Hospital.

### Screen Time Behaviour and Physical Activity

Time (hours per day) spent watching TV, playing seated (inactive) video games (excluding computer games), and recreational computer time (excluding school work) was assessed by self-report questionnaire. Total screen time was calculated by aggregating the three types of screen behaviours. Self-reported PA duration was calculated based on the question “On average, how long do you participate in some sort of physical activity each day – with physical activity being cumulative not consecutive.” Likert-type rating scales included 6 response ratings, whereby 1 = less than 5 minutes, 2 = 5 to 15 minutes, 3 = 15 to 30 minutes, 4 = 30 to 45 minutes, 5 = 45 to 60 minutes, and 6 = greater than 60 minutes. Intensity of PA was assessed by the question “on average, how would you describe the intensity of most of your physical activity?” Response ratings included 1 = light, 2 = moderate, and 3 = vigorous. Both PA duration and PA intensity were used as covariates in our statistical analyses.

### Lipids

Overnight-fasting blood samples of approximately 20 mL of venous blood were taken in the morning, from a forearm or antecubital vein and were transported directly to the laboratory at the Ottawa Hospital for analysis. The lipid profile measurements included triglycerides (mmol/L), total cholesterol (mmol/L), and HDL-C (mmol/L) which were measured by using enzymatic methods on a Beckman-Coulter LX20 analyzer (Beckman instruments, Brea, California), while LDL-C concentrations were calculated by using the Friedewald equation [27]. Total cholesterol/HDL-C ratio was derived from measured values.

### Blood Pressure

BP was measured manually using a mercury sphygmomanometer on the left arm after 4 minutes of rest, with subjects sitting with their back supported. Three BP measurements were taken at 1-minute intervals; the mean of the final two measures of BP was used for the analysis.

### Covariates

Weight and height were measured using a Health O Meter manual scale (Health O Meter, Continental Scale Corp, Bridgeview, ILL) while participants wore light clothing, without shoes, using standard techniques. BMI was calculated as weight (kg)/height in metres^2^. Children were classified as overweight or obese according to age and sex specific cut-points as described above in the participants section [26]. Sexual maturity was assessed using the 5 stage scale for breast development in females and testicular volume in males, according to Tanner [28].

Participants completed questionnaires on the same date as the physical measurements were taken. They were asked to report their age, sex, race and whether their mother and father completed elementary school, high school, community college or university. Highest level of maternal and paternal education was assessed as a proxy for socio-economic status.

Participant dietary intake, including total kilocalories consumed, kilocalories (and percent kilocalories) from fat, protein, and carbohydrates were assessed using 3-day, 24 hour food diaries under the supervision of a registered dietitian. The research coordinator provided instruction on food recording prior to evaluation of energy intake, and participants were provided with tools to aid in measurement. Food records were completed for three days, two weekdays and one weekend day, and the mean intake was averaged across the three days using food composition analysis software (The Food processor SQL 2006, ESHA Research, Salem, OR). A registered dietitian met with each participant shortly after completing the 3-day food records to clarify recording and gain more accurate measurement of energy intake.

### Statistical Analyses

The distributions for BP and lipid outcomes were examined to determine if they were normally distributed. All distributions were normally distributed except for triglycerides, which was positively skewed but normalized when it was logarithmically transformed, and this transformed triglycerides variable was used in the analyses. Sex differences were examined by independent t-tests and chi-squares for continuous and categorical variables, respectively (Table 1). The relationships between the covariates and the individual BP and lipid outcomes were assessed using Pearson Correlations for continuous variables and Spearman Rho (non-parametric) correlations for categorical variables (Table 2). The independent associations between the types of sedentary behaviour (seated video games, TV, recreational computer time, total screen time) and PA duration and PA intensity with BP and lipid measures (dependent variables) were tested by multiple linear regression, adjusting for sex, age, highest level of maternal and paternal education, sexual maturity, BMI, total caloric intake, percent of calories from dietary fat, PA intensity and PA duration (Table 3). Each regression for the associations between sedentary behaviours and BP and lipid outcomes were also assessed for a sex×sedentary behaviour interaction. Regarding the association between PA duration and PA intensity and BP and lipid outcomes, total screen time was used as the sedentary behaviour in all regressions because it represents an aggregate measure of the time spent in all screen time behaviours. For regressions involving PA duration and PA intensity, sex×PA interactions were also assessed for each BP and lipid outcome. Statistics were analyzed with SPSS for Windows (Version 18) and a p-value≤0.05 denoted statistical significance.

**Table 1:** Descriptive characteristics of the sample.

	Males (N = 86)	Females (N = 197)
Age (years)	15.4±1.3	15.6±1.4
Height (CM)***	174.5±7.6	165.4±5.9
Weight (kg)***	108.3±16.1	93.1±14.7
BMI (kg/m^2^)**	35.5±4.0	33.9±4.4
Tanner (pubertal) Stage***	4.5±0.5	4.8±0.4
TV viewing (hours/day)	2.7±1.7	2.8±2.1
Computer time (hours/day)	2.5±1.9	2.2±2.0
Video games (hours/day)***	1.5±1.9	0.2±0.6
Screen time (hours/day)***	6.7±2.9	5.2±2.8
Intake in Dietary Fat (%)	34.4±6.4	34.0±5.6
Total caloric intake (kcals)**	2322±601	2086±590
Physical activity score	3.5±1.7	3.4±1.7
PA intensity-light (%)	68.6	64.3
PA Intensity-moderate (%)	29.1	33.2
PA intensity-vigorous (%)	2.3	2.5
Systolic BP (mm Hg)***	119.0±10.9	111.3±9.1
Diastolic BP (mm Hg)	75.6±6.8	74.4±7.3
Triglycerides (mmol/L)*	1.4±0.6	1.2±0.6
HDL-C (mmol/L)***	1.0±0.2	1.1±0.3
LDL-C (mmol/L)	2.6±0.7	2.6±0.7
Total cholesterol (mmol/L)	4.2±0.8	4.3±0.8
Cholesterol/HDL-C Ratio***	4.4±1.2	3.9±1.0

Data are presented as means ± standard deviations except for PA intensity which are in percentages; PA = Physical activity score of 1 = less than 5 minutes, 2 = 5 to 15 minutes, 3 = 15 to 30 minutes, 4 = 30 to 45 minutes, 5 = 45 to 60 minutes, and 6 = Greater than 60 minutes. Sex differences determined by independent t-tests for continuous data and chi-square for categorical data;

*p<.05,

**p<.01,

***p<.001.

**Table 2:** Correlations between Various Sedentary Behaviours, Physical Activity and Blood Pressure and Lipids.

	TV	CT	VG	ST	BMI	Age	Sex	PE	ME	TS	%Fat	Cal	PA-I	PA-D
Systolic BP	0.06	−0.08	0.20**	0.07	0.25**	0.15**	−0.33**	0.06	0.01	0.01	0.10	0.10	0.12	0.03
Diastolic BP	0.00	−0.04	0.03	−0.02	0.25**	0.15**	−0.09	0.05	−0.01	0.01	0.08	0.06	−0.02	0.01
Total Cholesterol	0.03	−0.01	0.00	0.01	−0.05	0.07	0.04	−0.04	0.09	0.01	0.03	0.00	−0.05	−0.04
HDL-C	−0.03	0.08	−0.19**	−0.05	−0.25**	0.14**	0.27**	0.04	0.08	0.19**	−0.04	−0.08	0.10	0.04
Triglycerides	0.08	−0.09	0.10*	0.03	−0.23**	0.10*	−0.12*	−0.04	−0.01	−0.12*	0.07	0.04	−0.03	−0.05
LDL-C	0.02	−0.01	0.03	0.02	0.06	0.08	−0.01	−0.07	−0.01	−0.12*	0.03	0.02	−0.09	−0.04
Total Chol/HDL-C	0.04	−0.04	0.19**	0.08	0.25**	−0.07	−0.23**	−0.09	−0.01	0.15**	0.06	0.07	−0.11*	0.09

TV = television viewing in hours/day; CT = recreational computer time in hours/day; VG = video games in hours/day; ST = screen time in hours/day; Sex; 0 = male, 1 = females; ME = Maternal education; PE = paternal education; TS = tanner pubertal stage score; %Fat = %intake in dietary fat; Cal = total caloric intake(kcals); PA-I; Physical Activity Intensity, 1 = light, 2 = moderate, 3 = vigorous; PA-D = Physical Activity Duration score (hours/day). Pearson correlations used to assess continuous variables and Spearman Rho correlations used for categorical variables.

*p<.05;

**p<.001.

**Table 3:** Independent Associations of Various Sedentary Behaviours, Physical Activity Intensity and Blood Pressure and Lipids.

	TV	CT	VG	ST	PA- Intensity
Systolic BP	0.48 (−0.72 to 1.7)	−0.77 (−1.8 to 0.28)	1.1 (0.11 to 2.2)*	0.34 (−0.36 to 1.0)	−2.3 (−0.3 to −4.3)*
Diastolic BP	−0.09 (−0.21 to 0.03)	−0.02 (−0.12 to 0.09)	−0.01 (−0.12 to 0.09)	−0.04 (−0.12 to 0.03)	−0.58 (−2.1 to 0.96)
Total Cholesterol	−0.01 (−0.11 to 0.09)	−0.07 (−0.02 to 0.16)	0.03 (−0.06 to 0.17)	0.04 (−0.02 to 0.10)	−0.12 (−0.23 to 0.05)
HDL-C	0.00 (−0.03 to 0.03)	0.01 (−0.02 to 0.04)	−0.02 (−0.04 to 0.01)	0.00 (−0.02 to 0.02)	0.05 (0.00 to 1.0)*
Triglycerides	0.05 (−0.03 to 0.13)	−0.04 (−0.11 to 0.03)	0.04 (−0.03 to 0.11)	0.02 (−0.03 to 0.07)	−0.05 (−0.19 to 0.08)
LDL-C	0.03 (−0.12 to 0.05)	0.07 (−0.01 to 0.15)	0.02 (−0.05 to 0.10)	0.03 (−0.02 to 0.08)	−0.15 (−0.29 to −0.01)*
Total Chol/HDL-C	−0.03 (−0.16 to 0.11)	0.05 (−0.06 to 0.17)	0.10 (0.00 to 0.21)*	0.07 (−0.01 to 0.14)	−0.28 (−0.50 to −0.06)*

Data are presented as unstandardized βeta –Coefficients (95% confidence interval). βeta –Coefficients assess the relationship between the sedentary and physical activity variables (IVs) with the BP and lipid outcomes (DVs) adjusting for the covariates, thus the higher the beta weight, the stronger the independent relationship between the IVs and DVs; TV = television viewing in hours/day; CT = recreational computer time in hours/day; VG = video gameplaying in hours/day; ST = screen time in hours/day; PA-Intensity = Physical Activity Intensity score, with 1- light, 2 = moderate, 3 = vigorous.

*p<.05; Each linear regression assessing sedentary behaviours and PA intensity (primary IV's) on BP and lipids controlled for sex, mother and father's highest level of education, sexual maturity, age, BMI, total caloric intake (kcals) and % calories in dietary fat, physical activity duration and intensity and a sex×sedentary behaviour interaction. For the case of PA intensity, screen time was chosen as the sedentary behaviour because it is an aggregate measure of types of sedentary behaviour, and sex×PA intensity interaction was included in the regression equation. Results of regressions for PA duration and BP and lipids are presented in text of results section.

## Results

The descriptive characteristics of the sample are shown in Table 1. On average, males had a higher BMI, reported more time spent playing seated video games, greater total screen time, and greater caloric intake compared to females. Males were significantly more likely to be obese than females (100% vs 88%, p<0.001). Males also had higher systolic BP, triglycerides, and total cholesterol/HDL ratio, while females were more sexually mature and had higher HDL-C. There were no sex differences on self-reported PA.

Unadjusted correlations are shown in Table 2. None of the sedentary behaviours was significantly correlated with BP or lipids except for time spent playing video games, which was positively associated with systolic BP (r = 0.20, p<0.001), triglycerides (r = 0.10, p<0.001), total cholesterol/HDL-C ratio (r = 0.19, p<0.001), and negatively associated with HDL-C (r = −0.19, p<0.001). Although PA duration was not associated with any BP or lipid measures, PA intensity was inversely correlated with systolic BP (r = −0.12, p<0.05) and total cholesterol/HDL-C ratio (r = −0.11, p<0.05). There were no significant associations between total caloric intake (kcals) or percent fat intake and BP and lipids, as shown in Table 2. Similarly, there was no significant relationship between percent protein or carbohydrate intake and BP and lipids (data not shown). BMI correlated with BP and lipids more strongly than any other of the covariates.

Table 3 shows the independent associations between the types of sedentary behaviour and intensity of PA and BP and lipids after adjustment for potential confounders. The adjusted sex×sedentary behaviour interaction was not significant for any of the health outcomes. Similarly, the sex×PA duration and sex×PA intensity interactions were not significant (data not shown). However, after adjusting for age, sex, parental education, BMI, sexual maturity, total caloric intake, percent of energy intake derived from dietary fat, duration of PA, and intensity of PA, the positive associations between video gaming and systolic BP (adjusted r = 0.13, β = 1.1, p<0.05) and total cholesterol/HDL ratio (adjusted r = 0.12, β = 0.10, p<0.05) remained significant. The relationship between video gaming and triglycerides reached borderline significance (adjusted r = 0.11, β = 0.04, p = .07). No other types of sedentary behaviour, including total screen time, were associated with BP or lipid measures after adjusting for covariates. After adjustment, PA intensity was inversely associated with systolic BP (adjusted r = −0.13, β = −2.3, p<0.05), LDL-C (adjusted r = −0.12, β = −0.15, p<0.05), and total Cholesterol/HDL ratio (adjusted r = −0.14, β = −0.28, p<0.05), and positively associated with HDL-C (adjusted r = 0.11, β = 0.05, p<0.05). After adjustment, PA duration was only inversely associated with total cholesterol/HDL-C ratio (adjusted r = −0.11, β = −0.08, p<0.05).

## Discussion

This study examined the independent association between duration of screen time and time spent in types of sedentary screen behaviours with BP and lipids in overweight and obese adolescents. Interestingly, total screen time was not associated with BP or lipids before or after adjusting for confounders. However, to our knowledge, we are the first to show that time spent playing video games was the only type of sedentary behaviour associated with increased BP and lipids before and after adjusting for multiple confounders in a large sample of overweight and obese adolescents. Although males spent significantly more time playing video games than females, the sex×video game interaction was not significant, suggesting that the relationship between video games and BP and lipid outcomes did not differ by sex.

Only a few studies have examined the relationship between types of screen time behaviours and chronic disease risk factors in youth. In a large, nationally representative sample of over 2500 children and adolescents from the 2003/4 and 2005/6 National Health and Nutrition Examination Survey (NHANES), Carson and Janssen [24] found that time spent watching TV was predictive of a higher score of an aggregated or clustered measure of cardio-metabolic risk, but recreational computer time was not. Similarly, Martinez-Gomez et al. [25] found that TV viewing but not computer time was associated with increased BP in pre-pubertal children. However, neither study assessed time spent playing seated video games, so it is uncertain how this form of sedentary behaviour would have predicted chronic disease risk compared to other screen behaviours. In contrast to these findings, neither TV viewing nor recreational computer time was associated with BP or lipids in the current study, whether considering unadjusted or adjusted relationships. These discrepant findings are somewhat surprising since many studies have documented independent relationships between TV viewing and biochemical markers of metabolic and CVD risk in children and adolescents [12]–[14], [24], though not all have found these relationships [15]. The discrepant findings are unlikely to be due to differences in absolute time spent in either type of sedentary behaviour or variation since the mean values for time spent in TV and seated video gaming in the current study was similar, as was the variability. However, our sample was comprised of overweight and obese adolescents rather than a nationally representative sample [24], so it is possible these differences in sample characteristics could explain, in part, the discrepant findings.

Given the novelty of the findings, it is difficult to speculate what mechanisms may link video game use to high BP and more adverse lipid profiles; however, a few possible explanations are offered. Similar to TV viewing, observational studies showed an association between video game playing and obesity in youth [19]–[21]. Well-controlled crossover laboratory research indicates that video game playing was associated with an increase in spontaneous food intake of energy dense snack foods compared to resting conditions [22], [23] which may have an adverse impact on obesity, BP and lipid profiles. Even though various dietary measures (percent of calories from fat and total caloric intake) were adjusted for in this study, residual confounding may have still been present, although unlikely given we found no association between food intake and the BP and lipid measures. In addition, laboratory studies have shown that seated video game playing acutely causes increased heart rate, elevated systolic and diastolic BP, increased sympathetic tone and mental workload compared to rest [22], perhaps due to the excitement, stress and concentration required for effective gaming. Given that these associations between seated video gaming and BP occurred in laboratory settings, it is possible that these effects become exacerbated and more chronic when video games are played frequently over time as observed in our study. It is also possible that the self-reporting of gaming was more accurate than other screen behaviours since gaming may be played in discrete bouts that are more distinct and memorable, whereas it may be more difficult to accurately quantify time spent watching TV or computer use because they may be more susceptible to periodic interruptions.

Similar to previous studies [15], [16], [24], [29], PA intensity in this study, primarily that performed at moderate-to-vigorous-intensity, was associated with lower BP and more favourable lipid profile before and after adjustment for confounders, including sedentary behaviour. However, duration of PA, defined as time spent in PA per day, was not associated with BP or lipids before adjustment, and was only associated with total cholesterol/HDL-C ratio after adjustment, indicating that PA intensity rather than duration of PA may be more closely related to a lower BP and more favourable lipid profile in this population. In addition, these findings highlight the notion that sedentary behaviour and PA are distinct constructs that may have different mechanisms in how they relate to health outcomes [30].

### Limitations and Strengths

This study has several strengths and limitations. We utilized a sample of overweight and obese adolescents volunteering for exercise intervention, thus it is uncertain whether our findings can be generalized to overweight and obese adolescents in the community. In addition, time spent in screen time behaviours and PA duration and intensity were measured by self-report which may introduce inaccuracies and bias in youth, whereby PA is generally over reported and sedentary behaviour is under reported [31], and it is possible that objective measures of these behaviours may have resulted in a different pattern of results. Another limitation is the cross-sectional design, which limits the ability to make causal inferences about the relationships observed. Also, males spent more time video gaming than females, but males only comprised about 30% of the sample, thus it is possible that sex differences between seated video games and BP and lipid profiles may have been detected if the sample was more balanced on sex, thus future research is needed to test the veracity of this hypothesis. Regarding the imbalance in sex in the current study, it is possible that males perceive obesity to be a problem warranting intervention only at greater degrees of obesity, perhaps explaining, in part, that males tended to have more adverse BP and lipid profiles than females.

Strengths of this study include an assessment of the three primary forms of sedentary screen time behaviours (seated video games, TV and recreational computer time), whereas previous studies in the pediatric population examining the relationship between types of sedentary behaviour and risk factors of chronic disease only included TV and computer time [17], [24], [25]. Our study highlights the importance of measuring seated video gaming, which large surveys show has mass appeal to teenagers [18], especially males, and our data also reflect the popularity of video games given time spent in gaming was comparable to TV viewing. Our study is also the first to examine the associations between sedentary screen time behaviours and BP and lipid profiles in an overweight and obese adolescent sample, who is at increased risk of CVD and premature mortality in adulthood compared to their normal weight peers [8], [9]. Additionally, the present study included the broadest spectrum of BP and lipid measures as a proxy for assessing CVD risk factors compared to other studies [12]–[16]. Assessing the full spectrum of BP and lipids is important because these CVD risk factors in adolescence track into adulthood [7], and heart disease and stroke are still leading causes of morbidity and premature mortality [32]. Lastly, the current study included a comprehensive set of covariates that statistically controlled for several confounding variables, strengthening the internal validity of the findings.

### Conclusions

To the best of our knowledge, the present study is the first to demonstrate that seated video gaming is associated with increased BP and lipids in a sample of overweight and obese adolescents, after controlling for adiposity, caloric intake, dietary fat intake, PA duration and intensity and several other important confounders. Our results provide further support for the public health guidelines recommending that children and youth limit their sedentary behaviour, especially screen time behaviours [33], [34]. Our findings provide support for reducing time spent playing seated video games as a possible means to promote health and prevent CVD in this high risk group of overweight and obese adolescents. Future research is needed to first replicate these novel findings and subsequently aim to elucidate the mechanisms linking seated video gaming and elevated BP and lipids in a high risk sample of overweight and obese youth.
